# Performance Analysis and Improvement of WPAN MAC for Home Networks

**DOI:** 10.3390/s100402821

**Published:** 2010-03-29

**Authors:** Saurabh Mehta, Kyung Sup Kwak

**Affiliations:** UWB Wireless Communication Research Center, Inha University, Nam-Gu, Incheon 402-751, Korea; E-Mail: kskwak@inha.ac.kr

**Keywords:** MAC protocol, IEEE 802.15.3, backoff algorithm, performance analysis, home networking

## Abstract

The wireless personal area network (WPAN) is an emerging wireless technology for future short range indoor and outdoor communication applications. The IEEE 802.15.3 medium access control (MAC) is proposed to coordinate the access to the wireless medium among the competing devices, especially for short range and high data rate applications in home networks. In this paper we use analytical modeling to study the performance analysis of WPAN (IEEE 802.15.3) MAC in terms of throughput, efficient bandwidth utilization, and delay with various ACK policies under error channel condition. This allows us to introduce a K-Dly-ACK-AGG policy, payload size adjustment mechanism, and Improved Backoff algorithm to improve the performance of the WPAN MAC. Performance evaluation results demonstrate the impact of our improvements on network capacity. Moreover, these results can be very useful to WPAN application designers and protocol architects to easily and correctly implement WPAN for home networking.

## 1. Introduction

**Figure 1.**
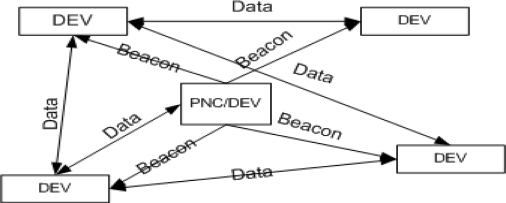
Piconet Structure in IEEE 802.15.3.

Recently, there is an increased need for high data rate wireless multimedia services such as uncompressed high-definition television (HDTV), instantaneous music, data, and video file transmission for both personal and home networking purposes. For this purpose, the IEEE 802.15.3 task group has been working on a promising standard including both medium access control (MAC) and physical layer (PHY), to realize short range and high data rate (1 Gbps or above) applications in WPAN. In this paper we discuss mainly about MAC layer issues in WPAN. The IEEE standard 802.15.3 MAC layer [1] is based on a centralized, connection oriented topology which divides a large network into several smaller ones termed as “piconets”. As shown in Figure 1, a piconet consists of a Piconet Network Controller (PNC) and DEVs (Devices). The DEV, a sensor node, is made to be low power and low cost. In a given piconet one DEV is required to perform the role of PNC (Piconet Coordinator), which provides the basic timing for the piconet as well as other piconet management functions, such as power management, Quality of Service (QoS) scheduling, and security. Using the formation of child and neighboring piconets users can increase the range of network span. The WPAN starter piconet is called a “parent piconet” and child/neighbor piconets are called “dependent piconets”. These piconets differ in the way they associate themselves to the parent piconet.

**Figure 2.**
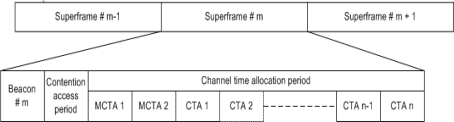
Superframe Structure of IEEE 802.15.3.

The IEEE 802.15.3 standard supports different power saving modes as well as multiple acknowledgement (ACK) policies (NO ACK, Imm-ACK, and Dly-ACK). IEEE 802.15.3 is very robust, stable and fast, and may coexist with other wireless technologies such as IEEE 802.11. In the IEEE 802.15.3 MAC protocol, at the start, communications are connection based under the supervision of PNC, however, at later stage connections and data transfer can be made in peer-to-peer fashion. In IEEE 802.15.3 MAC protocol, the channel time is divided into superframes, where each superframe beginning with a beacon. The superframe is made of the three major parts: the beacon, the optional contention access period (CAP), and the channel time allocation period (CTAP), as shown in Figure 2.

**Beacon**: The beacon is broadcast at the start of super frame by the PNC. All the information about timing allocation and management information are included in the beacon.
**Contention Access Period (CAP):** During the CAP period, DEVs can send command or asynchronous data packets using the CSMA/CA as the multiple access method.
**Channel Time Allocation Period (CTAP):** The channel time allocation period (CTAP) is composed of channel time allocations (CTAs), including management CTAs (MCTAs) and regular CTAs. This period is the same as the TDMA method and used to transmit commands, isochronous streams, and asynchronous data packets.

A wireless channel is usually vulnerable to errors. Hence, an error control mechanism is an essential part of any MAC protocol design. A good error control mechanism provides a certain level of reliability in terms of communication robustness and dependability for higher network layers. In accordance with that, IEEE 802.15.3 standard defines three types of acknowledgment mechanisms for CTAs and CAPs as follows:

**No-ACK:** In No-ACK (No-Acknowledgement) mechanism, ACK is not sent after a reception of message. This mechanism is useful for only high data rate applications where guaranteed delivery is not required or acknowledgement is handled by higher layers or by some other mechanism.
**Imm-ACK:** In Imm-ACK (Immediate Acknowledgement), mechanism, each received data frame is individually acknowledged after the successful reception. This mechanism provides simpler and more stable operation compared to No-ACK, but at the cost of reduced data rate.
**Dly-ACK:** In the Dly-ACK (delay-acknowledgement) mechanism, a receiver sends an acknowledge frame for a group of data frames rather than an individual data frame. This mechanism is a tradeoff between No-ACK and Imm-ACK. Both Imm-ACK and Dly-ACK have adopted a retransmission method to recover the corrupted frames in unsuccessful transmissions [1].

**Figure 3.**
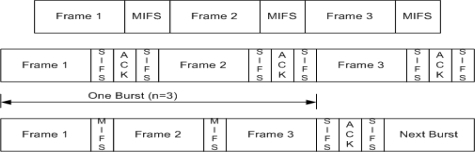
No-ACK, Imm-ACK and Dly-ACK (Burst Size = 3) : Different ACK Policies in IEEE 802.15.3.

In [2] and some other literature implied-acknowledgment (Imp-ACK) was proposed for bidirectional communication. Implied acknowledgement (Imp-ACK) permits a CTA to be used bi-directionally within a limited scope. During the CAP, Imp-ACK cannot avoid ambiguities between two frames; (a) the frame that is transmitted in response to a frame with an implied ACK request, and (b) the frame that is transmitted independently when the original frame is unsuccessfully received. In this paper we focused only on the three aforementioned acknowledgement schemes as Imp-ACK is currently neither widely accepted in the research literature nor in standard documents [1,2]. Figure 3 shows the different ACK policies in IEEE 802.15.3.

All these ACK policies have a large impact on the throughput, delay, and channel utilization of the network and require a detailed study to determine overall performance or channel capacity of the network. In this paper, we present a performance analysis of IEEE 802.15.3 from a protocol architecture’s point of view. Furthermore, we show the impact of our improvements in WPAN MAC. In a nutshell, the main contributions of this paper are as follows.

- To present an analytical model and performance evaluation study to determine optimization of payload size and ACK policies under error channel conditions.
- To introduce a Dly-ACK-AGG policy to improve the performance of WPANs.
- To apply payload size adjustment mechanism to improve the WPAN MAC under error channel conditions.
- To apply the Improved Backoff (IB) algorithm instead of the Binary Exponential Backoff (BEB) algorithm with CSMA/CA method during the CAP period.

## 2. Related Work

To the best of our knowledge, there is little published work on the performance or channel analysis of IEEE 802.15.3 MAC with respect to different ACK policies, under error channel conditions. However, a large amount of literature is available on IEEE 802.15.3 MAC scheduling, optimization of superframe size, and various traffic analyses. Some of the important related works are as follows.

In [3] the authors presented the implementation of IEEE 802.15.3 module in ns-2 and discussed various experimental scenario results, including various scheduling techniques, specifically, to investigate the performance of real-time and best-effort traffic with various super frame lengths and different ACK policies. In [4] the authors presented an adaptive Dly-ACK scheme for both TCP and UDP traffic with two main contributions. The first one is to request the Dly-ACK frame adaptively or change the burst size of Dly-ACK according to the transmitter queue status. The second is a retransmission counter to enable the destination DEV to deliver the MAC data frames to an upper layer in a timely and orderly fashion. In [5], its authors mainly focused on optimization of channel capacity. Both [4,5] lay a good foundation for simulation and analytical works on the IEEE 802.15.3 MAC protocol. Similarly, in [6] the authors formulated a throughput optimization problem under error channel conditions and derive a closed form solution for the optimal throughput. In [7] the authors presented a detailed study on performance analysis of Dly-ACK policy and proposed a dynamic Dly-ACK policy for WPAN MAC. Furthermore, they showed that the optimal burst size of Dly-ACK is heavily dependent on the input traffic load and is insensitive to the channel error rate within a normal error-rate change. The authors in [9] presented the throughput analysis of mm-wave WPAN and introduced a private channel release time mechanism to increase the throughput of mm-wave WPAN.

The work presented in [6] is close to our work, but their analysis scope is limited only in terms of throughput analysis, while our work span covers the delay, throughput, and channel utilization with different ACK policies under error channel conditions. Furthermore, we propose K-Dly-ACK-AGG policy, payload size adjustment mechanism, and Improved Backoff algorithm to improve the performance of the WPAN MAC.

The rest of the paper is organized as follows. In Section 3 we present the improvements in WPAN MAC and the design and analysis of the WPAN MAC in Section 4. Finally, conclusions are drawn in Section 5.

## 3. Improvements in WPAN MAC

In this section we introduce the following schemes to improve the performance of WPAN MAC for home networking.

### 3.1. K-Dly-ACK-AGG Policy

**Figure 4.**
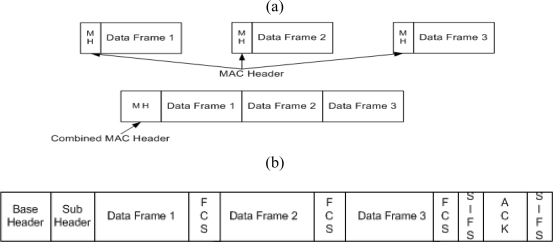
Aggregation Method and K-Dly-ACK-AGG Policy. (a) Aggregation Method; (b) K-Dly-ACK-AGG Policy (K = 3).

To reduce the overhead of the IEEE 802.15.3 MAC, we use the concept of frame aggregation. The idea of frame aggregation is to aggregate multiple MAC frames into a single (or approximately single) transmission [9], as shown in Figure 4(a).

In this paper we combine the frame aggregation concept and Dly-ACK mechanism with some modification and we define this new mechanism as K-Dly-ACK-AGG, as shown in Figure 4(b), where K is the burst size of data frames, [10]. Imm-ACK with aggregation method act same as K-Dly-ACK-AGG (where K = 1) so there is no point in considering Imm-ACK policy individually with aggregation. The main advantage of K-Dly-ACK-AGG policy is that it can make redundant inter-frame spaces and replace MAC headers with smaller size headers in Dly-ACK policy. This scheme is similar to the Blk-ACK policy in IEEE 802.15.3c. However, the Blk-ACK policy is only used with the aggregation whereby our K-Dly-ACK-AGG scheme does not have such a restriction. Furthermore, the K-Dly-ACK-AGG scheme offers much simpler ACK mechanism and frame format (it can easily switch between Dly-ACK, K-Dly-ACK-AGG, and Imm-ACK) [10].

### 3.2. Improved Backoff (IB) Algorithm

**Figure 5.**
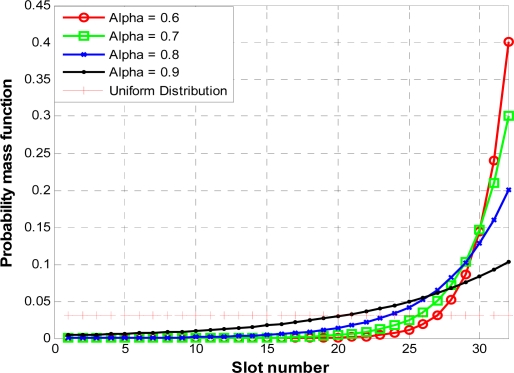
Difference between uniform and truncated geometric distributions.

In contrast to the BEB scheme, the IB scheme uses a small and fixed CW. In an IB scheme, nodes choose non-uniform geometrically increasing probability distribution (P) for picking a transmission slot in the contention window. Nodes which are executing the IB scheme pick a slot in the range of (1, CW) with a probability distribution P. Here, CW is the contention window and its value is fixed. We will present more information on CW in the later sections of this paper. Figure 5 shows the probability distribution P. The higher slot numbers have higher probability to get selected by nodes compared to lower slot numbers. In physical meaning we can explain this as: at the start node select a higher slot number for its CW by estimating large population of active nodes (n) and keep sensing the channel status. If no nodes transmit in the first or starting slots then each node adjusts its estimation of competing nodes by multiplicatively increasing its transmission probability for the next slot selection cycle. Every node keeps repeating the process of estimation of active nodes in every slot selection cycle and allows the competition to happen at geometrically–decreasing values of n all within the fixed contention window (CW).

In contrast to the probability distribution P, in uniform distribution, as shown in Figure 5, all the contending nodes have the same probability of transmitting in a randomly chosen time slot. From Figure 5 we can conclude that when the population of competing nodes (n) is large, most of the nodes will choose medium to high slot numbers as their CW and a very few nodes will choose low slot numbers, hence, a collision-free transmission will take place in a low slot number. When n is medium, most nodes will choose higher slot numbers and a collision-free transmission will take place in a medium slot number. Similarly, when n is small, a collision free transmission will take place in a high slot number. Thus for any value of n, and for any fast change in n, a collision free transmission can take place. If only one node gets the chance to select the contention slot within the fixed CW, it will transmit in that slot. Other nodes will select new random contention slots for the next competition to access channel medium, regardless of the success or failure of transmission of the winner node. Here, it is noteworthy that the IB scheme does not use timer suspension like in IEEE 802.11 to save energy and reduce latency in case of a collision. Also, we do not need to consider the fairness issue of IB here as every node does not have to request a packet for PNC every time during CAP. For more details readers are referred to [11].

### 3.3. Payload Adjustment Mechanism (PAM)

In this paper the channel bit error rate (BER), denoted as *p_e_*, (0 < *p_e_*< 1) can be calculated via previous frames or some other mechanism. How to obtain *p_e_* is beyond the scope of this paper. At higher values of BER the throughput performance of WPAN MAC decreases to a very low value. One way to counteract this problem is to reduce the size of payload with increasing BER value. So we apply a simple payload adjustment mechanism technique to WPAN MAC. This mechanism can control the size of the MAC protocol data unit (MPDU) based on the calculated value of BER. PAM is very simple to implement on the top of WPAN MAC without many changes to the existing protocol. From the results (see Figure 18) we can see the improvement in throughput performance with PAM over existing protocol.

## 4. WPAN MAC: Designing and Analysis

In this section, we present the design and analysis of IEEE 802.15.3 MAC to answer several questions like optimization of payload, optimization of ACK policies, and effect of aggregation, under various parameter conditions.

### 4.1. Approach and Assumptions

As we mentioned earlier most of the studies on WPAN MAC are focused on MAC scheduling, superframe optimization, etc., rather then performance analysis of WPAN MAC from a protocol architecture point of view. In this paper our main focus is to analyze and optimize the performance WPAN MAC with different ACK policies and proposed improvements to WPAN MAC under error channel conditions. Here, the optimization problem is defined as how to find the maximum throughput and channel utilization for a given channel bit error rate (BER) during the CTA/CAP period with various ACK policies under varying payload size. In this paper, we assume a Gaussian wireless channel model. Although the Gaussian channel model cannot capture the multi-path fading characteristics of a wireless channel, it is widely used because of its simplicity. The capture effect is also not considered. The Back off algorithm (During CAP period) performed in a time-slotted fashion. A node attempts to attain the access the channel only at the beginning of a slot. Furthermore, all nodes are well synchronized in time slots and propagation delay is negligible compared to the length of an idle slot. In the WPAN MAC, the channel time request command should be successfully transmitted between a node (in the remainder of the paper we use the terms ‘DEV’ and ‘node’ interchangeably) and the PNC to realize the communication during CTA. Usually, data transmission is performed during the CTAs and command transmission during the CAP. Here, for the simplicity we assume that a data frame is successfully transmitted if both data and ACK frames (except for No-ACK) are successfully received by receiver and transmitter, respectively. The analytical model presented in this paper does not depend on the technology adopted at the physical layer. However, the physical layer technology determines some network parameter values, e,g., SIFS, DIFS. Whenever necessary, we choose the values of these physical layer technology dependent parameters by referring to [16]. According to [16] we consider two different transmission rates for data signal and control signal, respectively.

### 4.2. Analytical Model

As we have shown in Figure 2. WPAN MAC is a hybrid MAC protocol based on the CSMA/CA method and TDMA scheme. There are number of papers on analytical models for CSMA/CA and TDMA. However these models cannot be directly adopted for the performance analysis of WPAN MAC because these models do not address the overall performance of both CSMA/CA and TDMA schemes, Thus, to study the performance analysis of WPAN MAC, we present an analytical model of WPAN MAC in two parts: CSMA/CA method during CAP and TDMA scheme (in this paper, we use the words ‘algorithm’, ‘scheme’, and ‘method’ interchangeably) during CTA period. We use the ground work of [6,12] and [13] to present our analytical model of CTA. Table 1 shows the notations used for our analytical model. Readers are advised to have a look at Table 1 when referring to equations for parameter notation.

The theoretical throughput is given by:

(1) $$\mathrm{Th}=\frac{\mathrm{Transmitted}\;\mathrm{Data}}{\mathrm{Transmission}\;\mathrm{Cycle}\;\mathrm{Duration}}$$

From [12], a frame with a length *L* in bits, the probability that the frame is successfully transmitted can be calculated as:

(2) $$p_{s}={\left(1-p_{e}\right)}^{L}$$

Similarly, we can define *p_s_* for different ACK mechanisms as follows (it is noteworthy that header—all kind of headers, trailers, beacons, etc. or control signals – and data frames are transmitted with different rates; generally, data rate is quite high compared to control signal, so we also considered two different signal rates for data and control signals, respectively, as listed in Table 2):

(3) $$\begin{array}{l} p_{s-\text{Imm-ACK}}={\left(1-p_{e}\right)}^{(L_{\mathrm{Data}}+L_{\mathrm{MAC}-H}+L_{\mathrm{ACK}-I\text{mm}})*8}\\ p_{s-\text{No-ACK}}={\left(1-p_{e}\right)}^{(L_{\mathrm{Data}}+L_{\mathrm{MAC}-H})*8}\\ p_{s-\text{Dly-ACK}}={\left(1-p_{e}\right)}^{(L_{\mathrm{Data}}+L_{\mathrm{MAC}-H}+L_{\mathrm{ACK}-\mathrm{Dly}})K*8}\\ p_{s-K-\text{Dly-ACK-AGG}}={\left(1-p_{e}\right)}^{(L_{\mathrm{Data}}+L_{\mathrm{MAC}-H}+L_{\mathrm{MAC}-\mathrm{Hs}}+L_{\mathrm{ACK}-\mathrm{DLy}}\;)K*8} \end{array}$$

Here, we use Imm-ACK, No-ACK, Dly-ACK, and K-Dly-AGG-ACK to denote the immediate acknowledgement, No acknowledgement, delay acknowledgement, and delay acknowledgement with aggregation, respectively. A successful transmission time during CTA is given by:

(4) $$T_{S-\mathrm{CTA}}=\{\begin{array}{ll} \left(T_{\mathrm{MFS}}+T_{\mathrm{Data}}+T_{\mathrm{MAC}–H}+T_{\mathrm{pre}}+T_{\mathrm{PHY}}\right) & \mathrm{for}\;\mathrm{No}–\mathrm{ACK}\\ \left(2*T_{\mathrm{SIFS}}+T_{\mathrm{Data}}+T_{\mathrm{MAC}–H}+2*T_{\mathrm{pre}}+T_{\mathrm{PHY}}+T_{\mathrm{MAC}–\mathrm{ACK}}+T_{\mathrm{PHY}–\mathrm{ACK}}+T_{\mathrm{Imm}–\mathrm{ACK}}\right) & \mathrm{for}\;\mathrm{Imm}–\mathrm{ACK}\\ \left(K(T_{\mathrm{MFS}}+T_{\mathrm{Data}}+T_{\mathrm{MAC}–H}+T_{\mathrm{pre}}+T_{\mathrm{PHY}})+T_{\mathrm{SIFS}}+T_{\mathrm{MAC}–\mathrm{ACK}}+T_{\mathrm{PHY}–\mathrm{ACK}}+T_{\mathrm{Dly}–\mathrm{ACK}}+T_{\mathrm{pre}}\right) & \mathrm{for}\;\mathrm{Dly}–\mathrm{ACK}\\ \left(K*T_{\mathrm{Data}}+T_{\mathrm{MAC}–H}+T_{\mathrm{MAC}–\mathrm{HS}}+2*T_{\mathrm{pre}}+T_{\mathrm{PHY}}+T_{\mathrm{SIFS}}+T_{\mathrm{MAC}–\mathrm{ACK}}+T_{\mathrm{PHY}–\mathrm{ACK}}+T_{K–\mathrm{Dly}–\mathrm{ACK}–\mathrm{AGG}}\right) & \mathrm{for}\;K–\mathrm{Dly}–\mathrm{ACK}–\mathrm{AGG} \end{array}$$

In Equation (4)*T_MAC-H_* and *T_Data_* are calculated as *T_MAC-H_* = *L_MAC-H_/C_rate_*, and *T_Data_* = *L_Data_/D_rate_*, respectively. *C_rate_* and *D_rate_* are the control signal rate and data signal rate (*D_rate_* >> *C_rate_*), respectively. Similarly, *T_pre_* and *T_PHY_* are calculated from [16]:

From Equations (1), (3) and (4) the throughput during CTA is given by:

(5) $${\mathrm{Th}}_{\mathrm{CTA}}=\{\begin{array}{ll} \frac{p_{s–\mathrm{No}–\mathrm{ACK}}\;L_{\mathrm{Data}}*8}{T_{s–\mathrm{CTA}}} & \mathrm{for}\;\mathrm{No}–\mathrm{ACK}\\ \frac{p_{s–\mathrm{Imm}–\mathrm{ACK}}\;L_{\mathrm{Data}}*8}{T_{s–\mathrm{CTA}}} & \mathrm{for}\;\mathrm{Imm}–\mathrm{ACK}\\ \frac{p_{s–\mathrm{Dly}–\mathrm{ACK}}\;K*\;L_{\mathrm{Data}}*8}{T_{s–\mathrm{CTA}}} & \mathrm{for}\;\mathrm{Dly}–\mathrm{ACK}\\ \frac{p_{s–K–\mathrm{Dly}–\mathrm{ACK}–\mathrm{AGG}}\;K*\;L_{\mathrm{Data}}*8}{T_{s–\mathrm{CTA}}} & \mathrm{for}\;K–\mathrm{Dly}–\mathrm{ACK}–\mathrm{AGG} \end{array}$$

Based on the analytical model presented in [14], the upper theoretical throughput limit during CTA is given by:

(6) $${\mathrm{Th}}_{\mathrm{UL}–\mathrm{CTA}}=\{\begin{array}{ll} \frac{K*L_{\mathrm{Data}}*8}{2*T_{\mathrm{PHY}}+2*T_{\mathrm{pre}}+T_{\mathrm{MIFS}}+T_{\mathrm{DIFS}}} & \mathrm{for}\;K–\mathrm{ACK}–\mathrm{AGG}\\ \frac{L_{\mathrm{Data}}*8}{T_{\mathrm{PHY}}+T_{\mathrm{pre}}+T_{\mathrm{MIFS}}+T_{\mathrm{DIFS}}} & \mathrm{for}\;\mathrm{No}–\mathrm{ACK}\\ \frac{K*L_{\mathrm{Data}}*8}{T_{\mathrm{PHY}}+T_{\mathrm{pre}}+T_{\mathrm{MIFS}}+T_{\mathrm{DIFS}}} & \mathrm{for}\;K–\mathrm{No}–\mathrm{ACK}–\mathrm{AGG} \end{array}$$

To demonstrate the effect of K-Dly-ACK and K-Dly-ACK-AGG on bandwidth utilization, we define a metric named maximum effective bandwidth (MEB), based on [7], which is a fraction of time the channel is used to successfully transmit data frames *versus* the total channel time. The maximum effective bandwidth utilization during a CTA/CAP slot is given by:

(7) $$\begin{array}{l} {\mathrm{MEB}}_{\mathrm{CTA}}=\{\begin{array}{ll} K.\frac{L_{\mathrm{Data}}p_{s–\mathrm{Dly}–\mathrm{ACK}}}{T_{s–\mathrm{CTA}}} & \mathrm{for}\;\mathrm{Dly}–\mathrm{ACK}\\ K.\frac{L_{\mathrm{Data}}p_{s–K–\mathrm{Dly}–\mathrm{ACK}–\mathrm{AGG}}}{T_{s–\mathrm{CTA}}} & \mathrm{for}–K–\mathrm{Dly}–\mathrm{ACK}–\mathrm{AGG} \end{array}\\ {\mathrm{MEB}}_{\mathrm{CAP}}=\{\begin{array}{ll} K.\frac{L_{\mathrm{Data}}n\psi {\left(1-\psi \right)}^{n-1}p_{s–\text{Dly}–\text{ACK}}}{T_{s–\mathrm{CAP}}} & \mathrm{for}\;\mathrm{Dly}–\mathrm{ACK}\\ K.\frac{L_{\mathrm{Data}}n\psi {\left(1-\psi \right)}^{n-1}p_{s–K–\mathrm{Dly}–\mathrm{ACK}–\mathrm{AGG}}}{T_{s–\mathrm{CAP}}} & \mathrm{for}\;K–\mathrm{Dly}–\mathrm{ACK}–\mathrm{AGG} \end{array} \end{array}$$

**Figure 6.**
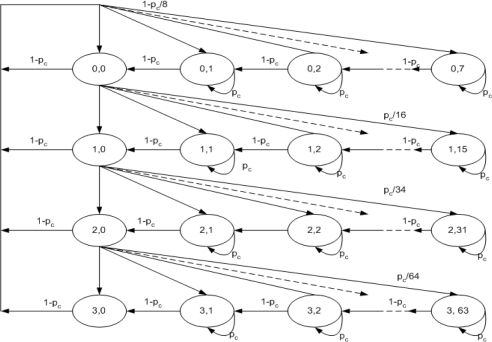
Markov Chain Model for IEEE 802.15.3.

During the CAP, the MAC protocol performs a back off procedure before transmitting any kind of data or request packets. This backoff mechanism is similar to the CSMA/CA mechanism of IEEE 802.11 with some different parameters. In WPAN MAC, the retry count is limited to a maximum of 3 counts (0 to 3) with maximum contention window (CW) size of 64 slots (8, 16, 32, and 64). After selecting CW node decrement, its value by 1 as long as the channel is sensed as idle and it freeze its value when the channel is sensed as busy. During CAP, if the Imm-ACK mechanism is used, every node acquires CSMA/CA with binary exponential backoff. During NO-ACK mechanism every node starts with some fixed backoff window value without any knowledge of success/failure of transmitted data frames. When the Dly-ACK mechanism is used, a node will randomly select some CW value and send a number (*K*) of data frames each separated by an MIFS with Dly-ACK request information in MAC header once its backoff timer reaches zero, and will wait for an ACK, as shown in Figure 3. If a burst transmission of *K* data frames is assumed to be successful, then the sender will reset the backoff window to the initial value; otherwise, the backoff window will be doubled. *K*-Dly-ACK-AGG follows the same backoff procedure as Dly-ACK. We model the operation of BEB at an individual node using the state diagram shown in Figure 6. This diagram is based on the model presented in [11,13] including the freezing and retry limit parameters.

As shown in the Figure 6 let j denote the backoff stage, where *j* = 0, 1, 2, 3. So, we have *CW*_0_ = 8, *CW*_1_ = 16, *CW*_2_ = 32, and *CW*_3_ = 64. Let *b(t)* be defined as a random process representing the backoff counter of a node and *s*(*t*) is representing random process of the back stage *j*. The term *b(t)* is decreased at the start of every idle backoff slot. It is important to note that the time scale for *b*(*t*) doesn’t represent real time but it observes only backoff slots and its suspended for the duration of all transmissions and interframe spaces (*i.e.*, SIFS). Whenever *b(t)* reaches zero the station transmits and regardless of the outcome of the transmission, uniformly chooses a new value for *b(t)* from (0,1,…,*CW_j_* − 1) (*i.e.*, a new backoff counter value). Here, we define *p_c_* as the conditional collision probability and we also assume that it is independent and constant, regardless the number of retransmissions attempted. *P*_c_ also represents the probability of detecting the channel busy. Thus, the two dimension process, {*s*(*t*),*b*(*t*)}, is a discrete-time Markov Chain. Therefore, the state of each node is described by {*j*,*k*}, where *j* stands for the backoff stage, and *k* stands for the backoff timer value. The state transition diagram of the Markov chain model shown in Figure 6 has the following transition probabilities:

(8) $$\{\begin{array}{lll} P\{j,k|j,k+1\}=(1-p_{c}) & k\in (0,{\mathrm{CW}}_{j}-2) & j\in (0,3)\\ P\{j,k|j,k\}=p_{c} & k\in (1,{\mathrm{CW}}_{j}-1) & j\in (0,3)\\ P\{j,k|j-1,0\}=p_{c}/{\mathrm{CW}}_{j} & k\in (1,{\mathrm{CW}}_{j}-1) & j\in (1,3)\\ P\{m,k|m,0\}=p_{c}/{\mathrm{CW}}_{j} & k\in (1,{\mathrm{CW}}_{j}-1) & \end{array}$$

The first equation in (8) indicates that at the beginning of each slot time, the backoff counter is decreased if the channel is sensed as idle. The second equation shows that the backoff counter is frozen if channel is sensed as busy. The third and fourth equations, respectively, indicate that following an unsuccessful transmission, the node back off stage (*j* − 1) selects a backoff interval uniformly in the range of (0,*CW_j_* − 1) and when the backoff stage reaches m, *CW_m_* stays constant. From the given backoff algorithm framework we can calculate the failure probability, the success probability, and the busy probability of a transmission during CAP. Here we derive throughput, efficient bandwidth utilization, and delay with these three basic probabilities. From [11,13], the failure probability of a transmission during CAP is given by:

(9) $$p_{c}=\{\begin{array}{ll} 1-(1-p)p_{s–\mathrm{Imm}–\mathrm{ACK}} & \mathrm{for}\;\mathrm{Imm}–\mathrm{ACK}\\ 1-(1-p)p_{s–\mathrm{Dly}–\mathrm{ACK}} & \mathrm{for}\;\mathrm{Dly}–\mathrm{ACK}\\ 1-(1-p)p_{s–K–\mathrm{Dly}–\mathrm{ACK}–\mathrm{AGG}} & \mathrm{for}\;K–\mathrm{Dly}–\mathrm{ACK}–\mathrm{AGG} \end{array}$$

where *p*, the probability of a transmitted frame collision for *n* number of station is given by:

(10) $$p=1-{\left(1-\psi \right)}^{n-1}$$

where *ψ*, probability of a station to transmit during a generic (*i.e.*, randomly chosen) ‘slot time’ is also depends on number of retry limit. This ‘slot time’ is contention window slot and it is different from the data transmission slot. Usually, data transmission slot is quite long compared to contention window slot. Then, the probability of the busy channel is given by:

(11) $$p_{b}=1-{\left(1-\psi \right)}^{n}$$

From Equations (10) and (11), the probability of a successful transmission occurs in a slot time is given by:

(12) $$p_{s}=\{\begin{array}{ll} n\psi {\left(1-\psi \right)}^{n-1}p_{s–\mathrm{No}–\mathrm{ACK}} & \mathrm{for}\;\mathrm{No}–\mathrm{ACK}\\ n\psi {\left(1-\psi \right)}^{n-1}p_{s–\mathrm{Imm}–\mathrm{ACK}} & \mathrm{for}\;\mathrm{Imm}–\mathrm{ACK}\\ n\psi {\left(1-\psi \right)}^{n-1}p_{s–\mathrm{Dly}–\mathrm{ACK}} & \mathrm{for}\;\mathrm{Dly}–\mathrm{ACK}\\ n\psi {\left(1-\psi \right)}^{n-1}p_{s–K–\mathrm{Dly}–\mathrm{ACK}–\mathrm{AGG}} & \mathrm{for}\;K–\mathrm{Dly}–\mathrm{ACK}–\mathrm{AGG} \end{array}$$

A successful transmission time during CAP is given by:

(13) $$T_{s–\mathrm{CAP}}=\{\begin{array}{ll} \left(\bar{\mathrm{CW}}+T_{\mathrm{MFS}}+T_{\mathrm{Data}}+T_{\mathrm{MAC}–H}+T_{\mathrm{pre}}+T_{\mathrm{PHY}}\right) & \mathrm{for}\;\mathrm{No}–\mathrm{ACK}\\ \left(\bar{\mathrm{CW}}+2*T_{\mathrm{SIFS}}+T_{\mathrm{Data}}+T_{\mathrm{MAC}–H}+2*T_{\mathrm{pre}}+T_{\mathrm{PHY}}+T_{\mathrm{MAC}–\mathrm{ACK}}+T_{\mathrm{PHY}–\mathrm{ACK}}+T_{\mathrm{Imm}–\mathrm{ACK}}\right) & \mathrm{for}\;\mathrm{Imm}–\mathrm{ACK}\\ \left(\bar{\mathrm{CW}}+K(T_{\mathrm{MFS}}+T_{\mathrm{Data}}+T_{\mathrm{MAC}–H}+T_{\mathrm{pre}}+T_{\mathrm{PHY}})+T_{\mathrm{SIFS}}+T_{\mathrm{MAC}–\mathrm{ACK}}+T_{\mathrm{PHY}–\mathrm{ACK}}+T_{\mathrm{Dly}–\mathrm{ACK}}+T_{\mathrm{pre}}\right) & \mathrm{for}\;\mathrm{Dly}–\mathrm{ACK}\\ \left(\bar{\mathrm{CW}}+K*T_{\mathrm{Data}}+T_{\mathrm{MAC}–H}+T_{\mathrm{MAC}–\mathrm{HS}}+2*T_{\mathrm{pre}}+T_{\mathrm{PHY}}+T_{\mathrm{SIFS}}+T_{\mathrm{MAC}–\mathrm{ACK}}+T_{\mathrm{PHY}–\mathrm{ACK}}+T_{K–\mathrm{Dly}–\mathrm{ACK}–\mathrm{AGG}}\right) & \mathrm{for}\;K–\mathrm{Dly}–\mathrm{ACK}–\mathrm{AGG} \end{array}$$

Here, *CW* represents the average back-off time. The average back-off defines the back-off duration for “light loaded networks”, *i.e.*, when each station has access to the channel after the first back-off attempt and is given by:

(14) $$\bar{\mathrm{CW}}=\frac{{\mathrm{CW}}_{\text{min}}.T_{\mathrm{slot}}}{2}$$

A failure transmission time during CTA is given by:

(15) $$T_{f–\mathrm{CTA}}=\{\begin{array}{ll} \left(T_{\mathrm{MFS}}+T_{\mathrm{Data}}+T_{\mathrm{MAC}–H}+T_{\mathrm{pre}}+T_{\mathrm{PHY}}\right) & \mathrm{for}\;\mathrm{No}–\mathrm{ACK}\\ \left(T_{\mathrm{SIFS}}+T_{\mathrm{Data}}+T_{\mathrm{MAC}–H}+T_{\mathrm{pre}}+T_{\mathrm{PHY}}+T_{\mathrm{ACK}–\mathrm{To}}\right) & \mathrm{for}\;\mathrm{Imm}–\mathrm{ACK}\\ \left(K(T_{\mathrm{MIFS}}+T_{\mathrm{Data}}+T_{\mathrm{MAC}–H}+T_{\mathrm{pre}}+T_{\mathrm{PHY}})+T_{\mathrm{ACK}–\mathrm{To}}+T_{\mathrm{SIFS}}\right) & \mathrm{for}\;\mathrm{Dly}–\mathrm{ACK}\\ \left(K*T_{\mathrm{Data}}+T_{\mathrm{MAC}–H}+T_{\mathrm{MAC}–\mathrm{HS}}+T_{\mathrm{pre}}+T_{\mathrm{PHY}}+T_{\mathrm{ACK}–\mathrm{To}}+T_{\mathrm{SIFS}}\right) & \mathrm{for}\;K–\mathrm{Dly}–\mathrm{ACK}–\mathrm{AGG} \end{array}$$

From Equations (12), (13), and (15), the throughput during CAP is given by:

(16) $${\mathrm{Th}}_{\mathrm{CAP}}=\{\begin{array}{ll} \frac{P_{S}L_{\mathrm{Data}}*8}{(1-p_{b})\delta +P_{S}T_{s–\mathrm{CAP}}+(p_{b}-P_{S})T_{f–\mathrm{CAP}}} & \mathrm{for}\;\mathrm{No}–\mathrm{ACK}\\ \frac{P_{S}L_{\mathrm{Data}}*8}{(1-p_{b})\delta +P_{S}T_{s–\mathrm{CAP}}+(p_{b}-P_{S})T_{f–\mathrm{CAP}}} & \mathrm{for}\;\mathrm{Imm}–\mathrm{ACK}\\ \frac{P_{S}K*L_{\mathrm{Data}}*8}{(1-p_{b})\delta +P_{S}T_{s–\mathrm{CAP}}+(p_{b}–P_{S})T_{f–\mathrm{CAP}}} & \mathrm{for}\;\mathrm{Dly}–\mathrm{ACK}\\ \frac{P_{S}K*L_{\mathrm{Data}}*8}{(1-p_{b})\delta +P_{S}T_{s–\mathrm{CAP}}+(p_{b}-P_{S})T_{f–\mathrm{CAP}}} & \mathrm{for}\;K–\mathrm{Dly}–\mathrm{ACK}–\mathrm{AGG} \end{array}$$

From [14], the upper theoretical throughput limit during CAP is given by:

(17) $${\mathrm{Th}}_{\mathrm{UL}–\mathrm{CAP}}=\{\begin{array}{ll} \frac{K*L_{\mathrm{Data}}*8}{\bar{\mathrm{CW}}+2*T_{\mathrm{PHY}}+2*T_{\mathrm{pre}}+T_{\mathrm{MIFS}}+T_{\mathrm{DIFS}}} & \mathrm{for}\;K–\mathrm{ACK}–\mathrm{AGG}\\ \frac{L_{\mathrm{Data}}*8}{\bar{\mathrm{CW}}+T_{\mathrm{PHY}}+T_{\mathrm{pre}}+T_{\mathrm{MIFS}}+T_{\mathrm{DIFS}}} & \mathrm{for}\;\mathrm{No}–\mathrm{ACK}\\ \frac{K*L_{\mathrm{Data}}*8}{\bar{\mathrm{CW}}+T_{\mathrm{PHY}}+T_{\mathrm{pre}}+T_{\mathrm{MIFS}}+T_{\mathrm{DIFS}}} & \mathrm{for}\;K–\mathrm{No}–\mathrm{ACK}–\mathrm{AGG} \end{array}$$

From (1), we can also calculate the average upper limit on access delay during CTA/CAP.

### 4.3. Performance Evaluation

In this subsection we present the performance evaluation of WPAN MAC in terms of throughput, efficient bandwidth utilization, and delay with different ACK policies under error channel conditions. For the performance evaluation we carried out a simulation in Matlab [15]. The main parameters for our simulation are based on [16] and listed in Table 2. For the simulation results we do not consider the technology adopted at the physical layer, however the physical layer determines some network parameter values like inter-frame spaces, *etc.* Whenever necessary we choose the values of the physical layer dependent parameters by referring to [16]. Also, we do not consider any specific scheduling algorithm to allocate the channel time slots as it is outside the scope of this paper. The design of a simple but effective scheduling algorithm is still an open issue. The results obtained here are the average values of our collected data.

**Figure 7.**
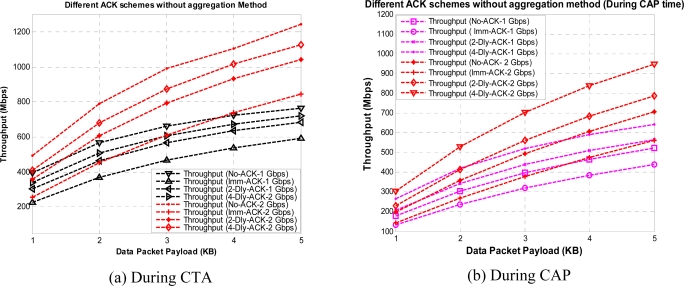
Throughput *versus* payload size with different ACK policies without aggregation during CTA and CAP.

Figure 7 shows the throughput for different payload size with different ACK polices without aggregation, for CTA and CAP, respectively. We assume an ideal channel condition for these results. Here, we can observe that No-ACK gives the best results as most of the CTA and CAP time is utilized for data transfer. However, the No-ACK policy is not suitable for every application due to its limited communication reliability, compared to other ACK policies. For a WPAN designer the design of a system system with higher data rate or higher throughput is always a key demand. One way to achieve this requirement is to increase the data rate; however, that also has some limitations.

**Figure 8.**
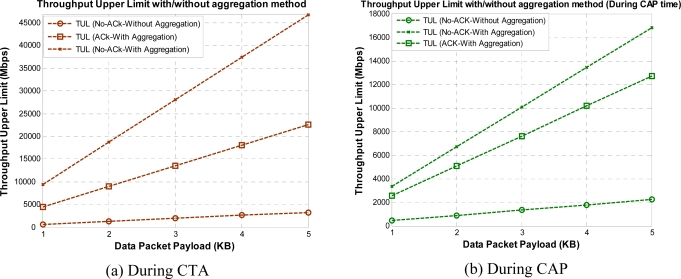
Throughput upper limit *versus* payload size during CTA and CAP.

**Figure 9.**
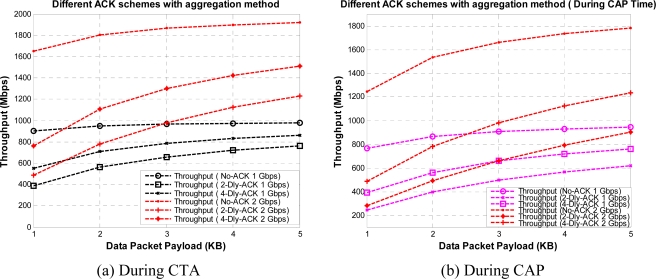
Throughput *versus* payload size with different ACK policies with aggregation during CTA and CAP.

Figure 8 shows the theoretical upper limit that exists on throughput. Even if we increase the data rate to infinite without reducing the overhead, we can only get achieve the bounded throughput, as shown in Figure 8. To reduce the overhead and to increase the throughput, with maximum available practical data rate [16], we adopt the frame aggregation method for WPAN. Now, Figure 9 shows the similar results but with aggregation method applied to different ACK policies in CTA and CAP, respectively. Here, No-ACK with aggregation (No-ACK-AGG) is nothing but the simple frame aggregation technique with maximum K burst size (K = 16) [10].

The K-Dly-ACK-AGG policy (in the remainder of the paper we use the terms “*K*-Dly-ACK-AGG” and “DLY-ACK with aggregation” interchangeably) can achieve somewhat close results to the No-ACK policy, as it reduces the unnecessary inter-frame time as well as the header size. For Figures 7(b) and 9(b) we assume a light load network, as our main focus is to get maximum throughput for each payload size. We also set contention window at its minimum value.

**Figure 10.**
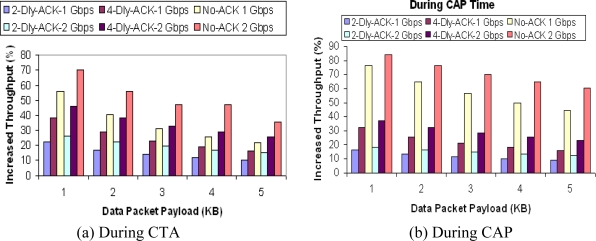
Increment in throughput.

From the aforementioned results it is easy to conclude that the aggregation method gives higher throughput at high data size but we found it to be the other way around. Figure 10 shows the percentage gain in throughput using an aggregation method for different payload sizes for CTA and CAP, respectively. We can observe that as payload size increases, percentage gain in throughput reduces, the reason for is being that at lower payload size we can send more data packets in given a CTA/CAP duration but this also increases overhead. With aggregation methods we can reduce overhead to a large extent but can’t get the same benefit at higher payload size. Thus there is an open issue for a designer to choose an appropriate data packet size for the needed gain in throughput.

**Figure 11.**
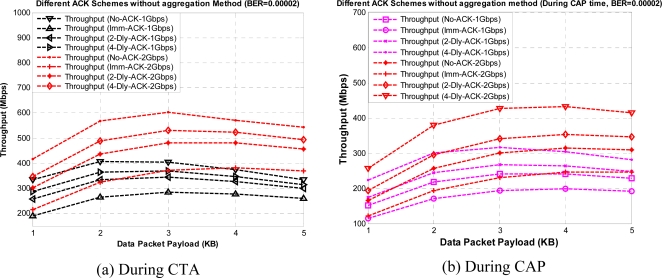
Throughput *versus* payload size with different ACK policies without aggregation during CTA and CAP.

**Figure 12.**
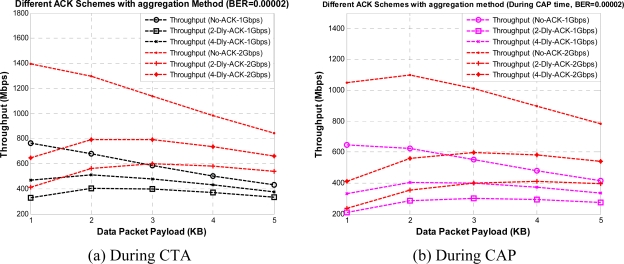
Throughput *versus* payload size with different ACK policies with aggregation during CTA and CAP.

From Figures 7 and 9 we can observe the value of throughput at different payload sizes but still these results are not sufficient to find the optimum payload size with given ACK policies. So, we obtained the same results under Gaussian wireless channel model with different BER rates. However, only a part of the results are presented here to reduce the number of graphs in order to maintain the lucidity of the paper. Figures 11 and 12 show the throughput for different payload sizes under a given BER value during CTA and CAP, with and without aggregation, respectively.

It can be seen that an optimal payload size exists for a given BER value, and the optimal payload size increases as BER values decreases. As shown in the mentioned figures the throughput first increases, and then decreases with increasing payload size (even with the aggregation) in error prone channels. This is because without the protection of FCS in an individual payload frame, a single bit error may corrupt the whole frame which will waste lots of medium time usage and counteract the efficiency produced by an increased payload size. So, the initial increase of the curves in figures show the effect of increased transmission efficiency over the effect of increased frame error probability, while decreses in the curves show the opposite results. From the above mentioned figures we can determine the optimum payload size value for a given BER value. As shown in the results K-Dly-ACK with aggregation policy give the best results after No-ACK policy. For all our throughput results during CAP time we selected a light-load network scenario. However, it is very interesting to note that for the duration of CAP duration [Figures 11(b) and 12(b)]; throughput performance depends on the number of active stations and backoff window size.

**Figure 13.**
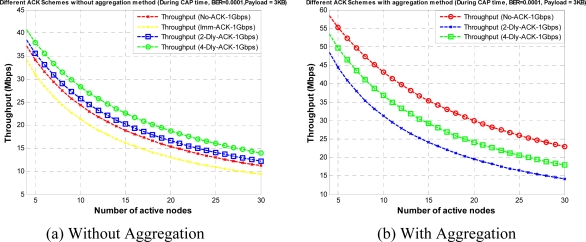
Throughput *versus* number of active nodes.

Figure 13 further investigates this observation. As shown in this figure the throughput under a given BER value, with and without aggregation, respectively, decreases as the number of active nodes increases due to corresponding increases in collision probability and channel access time. To counteract increasing in collision probability and channel access time we apply IB algorithm as backoff during the CAP, as explained in Section 3.

**Figure 14.**
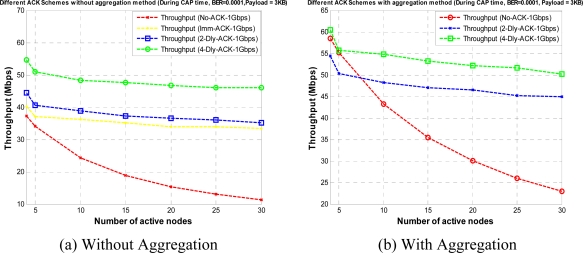
Throughput *versus* number of active nodes with IB as a backoff algorithm.

**Figure 15.**
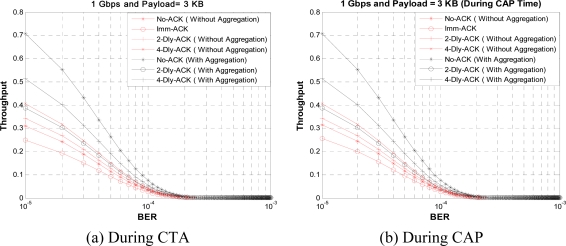
Throughput *versus* BER value with different ACK policies.

As shown in Figure 14, IB can improve the performance to a large extent because of its unique feature of avoiding collision among the competing nodes. Here we did not apply IB for No-ACK policy as it has to use with fixed CW size, so the results for No-ACK policy are same as shown in Figure 13. From the aforementioned results we only get the optimal payload size for a fixed BER value, which might not be a sufficient result for a WPAN designer, so to check the performance of a given network under the range of BER values with different payload sizes, we obtained the subsequent results as shown in Figure 15. As we mentioned earlier, for the sake of clarity in the paper, we omitted here several results with different payload sizes.

Figure 15 shows the normalized throughput for a given BER value with different ACK policies when payload size is set to 3 KB in CTA and CAP, respectively. As the BER value increases the optimal payload size and the optimal throughput decreases. From the figure we can observe that the No-ACK policy with aggregation has larger throughput over large range of BER values than other ACK policies, because a No-ACK policy with aggregation gives maximum usage of CTA and CAP duration. Also, we can notice that at higher value of BER throughput reaches to zero, at this value payload size is too big for the given system. To control the decrement in throughput we apply a payload size adjusting mechanism or payload adjusting mechanism (PAM) to WPAN MAC, as explained in Section 3.2. Along with an optimum payload size it is also important for a WPAN designer to find an optimum K burst size for frame aggregation policy, so to find the effect of K-Dly-ACK on bandwidth utilization as well as to find the optimal value of burst size for K-Dly-ACK and K-Dly-ACK-AGG policies, we define the MEB metric in (7). Here, Imm-ACK is a special case of K-Dly-ACK (where K = 1) so we do not need to define it separately.

**Figure 16.**
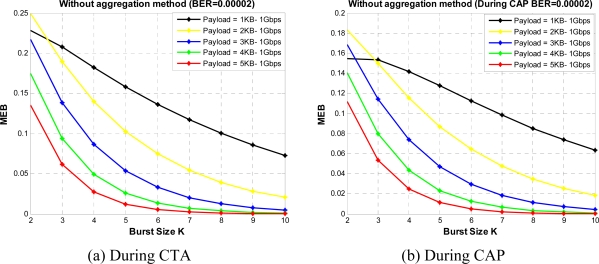
MEB *versus* burst size without aggregation.

**Figure 17.**
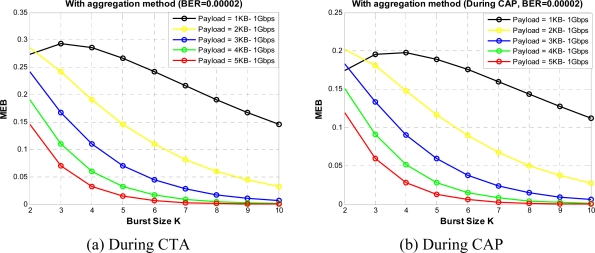
MEB *versus* burst size with aggregation.

**Figure 18.**
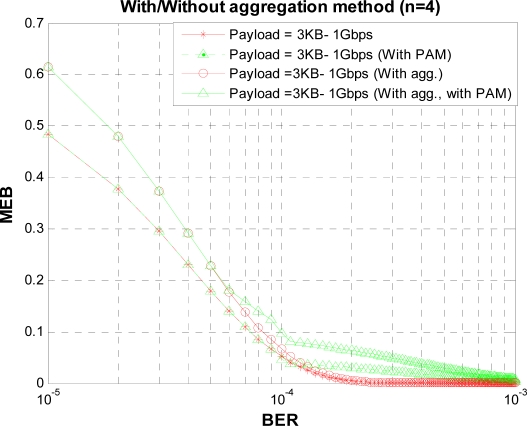
MEB *versus* BER value.

Figures 16 and 17 shows the MEB for different burst values for a given BER value in CTA and CAP, respectively. From the figures we see that when the burst size increases, bandwidth utilization can be increased initially, but the BER probability also increases and so the bandwidth utilization. From the figures we can find the optimum value for K for different payload sizes under a given BER value, From these figures we can observe that burst size K = 4 gives good results in fairly all payload values. So we select K = 4 and obtain the results for a range of BER values *versus* MEB as shown in Figure 18. As shown in Figure 18, we compare two sets of results: one with PAM and second without PAM. As we mentioned earlier, payload adjustment mechanism (PAM) can adjust the payload size according to the observed value of BER. After a certain threshold BER value PAM can set the payload size to a smaller value. In our simulations we set several threshold BER values for PAM to adjust the payload size which might not be optimized values. However, our results show that the PAM can have noticeable impact on MEB. In the future version of this paper we want to consider dynamic payload adjustment mechanism.

Here, the aggregation method clearly shows its advantage over the non aggregation method even at higher values of BER. Again it is an open tradeoff between MEB and payload size that a WPAN designer will have to decide according to his application requirements. This result also supports the need for PAM in WPAN MAC design.

**Figure 19.**
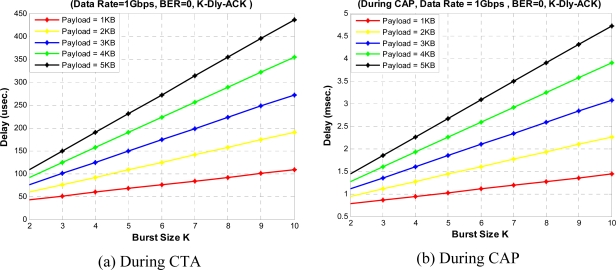
Access delay *versus* burst size.

Figure 19 shows the access delay performance for different burst sizes with aggregation method in CTA and CAP, respectively. Here, we define the access delay as the time from the moment a packet is ready to be transmitted to the moment the packet starts its successful transmission. For a WPAN designer it is very important to know the maximum possible delay limit for a given network. *K*-Dly-ACK-AGG policy gives the maximum delay limit compared to other ACK policies as it transmit large payload size with aggregation. Figure 19(a) shows access delay during CTA period where we do not need to consider any backoff and channel access delay, however, during CAP period, the obtained results [Figure 19(b)] are largely depends on fixed back off window size of the IB. As the number of burst size increases, access delay also increases linearly with it.

## 5. Conclusions

In this paper, we have studied the performance of WPAN MAC in terms of throughput, efficient bandwidth utilization, and delay with various ACK policies under error channel conditions. From the performance analysis we can determine the optimal payload size, burst size, and ACK policy for a given set of parameters. In order to improve the performance of WPAN we introduced the K-Dly-ACK-AGG policy, payload size adjustment mechanism, and Improved Backoff algorithm. Numerical results show that the proposed methods significantly improve the performance of the WPAN MAC. Finally, we hope that the results of this paper will help WPAN application designers and protocol architects to easily and correctly implement the WPAN networks based on IEEE 802.15.3 MAC technology.

## Figures and Tables

**Table 1.** Parameter Notations.

*T_SIFS_*	Short Inter Frame Space (SIFS) time
*T_DIFS_*	Distributed Coordinate Function Inter Frame Space (DIFS)
*T_MIFS_*	Minimum Inter Frame Space (MIFS) time, Usually MIFS ≤
*CW*_min_	Minimum back-off window size
*T_pre._*	Transmission time of the physical preamble
*T_PHY_*	Transmission time of the PHY header
*L_MAC–H_*	MAC overhead in bytes
*L_ACK_*	ACK size in bytes
*L_Data_*	Payload size in bytes
*T_MAC–H_*	Transmission time of MAC overhead
*T_ACK_*	ACK transmission time
*T_Data_*	Transmission time for the payload
*T_f–CAP_*	The time for a transmission considered failed during CAP
*T_s–CAP_*	The time for a transmission considered successful during CAP
*T_f–CTA_*	The time for a transmission considered failed during CAT
*T_s–CTA_*	The time for a transmission considered successful during CAT
*T_ACK–TO_*	The time-out value waiting for an ACK

**Table 2.** Parameters for Performance Evaluation.

**Parameters**	**Values**
SIFS	2.5 usec
MIFS	1 usec
Preamble and PLCP Header	9 usec
*CW*_min_	8
Payload Size	1 ∼ 5 KB
ACK Policy	3 basic + K-Dly-ACK-AGG
Data Rate	1 ∼ 2 Gbps
Control Signal Rate	48 Mbps
Nodes	1 ∼ 30
